# Development of an efficient technique for gene deletion and allelic exchange in *Geobacillus* spp.

**DOI:** 10.1186/s12934-017-0670-4

**Published:** 2017-04-05

**Authors:** Leann F. Bacon, Charlotte Hamley-Bennett, Michael J. Danson, David J. Leak

**Affiliations:** Department of Biology and Biochemistry, University of Bath, Bath, BA2 7AY UK

## Abstract

**Background:**

*Geobacillus thermoglucosidasius* is a thermophilic, natural ethanol producer and a potential candidate for commercial bioethanol production. Previously, *G. thermoglucosidasius* has been genetically modified to create an industrially-relevant ethanol production strain. However, creating chromosomal integrations and deletions in *Geobacillus* spp. is laborious. Here we describe a new technique to create marker-less mutations in *Geobacillus* utilising a novel homologous recombination process.

**Results:**

Our technique incorporates counter-selection using β-glucosidase and the synthetic substrate X-Glu, in combination with a two-step homologous recombination process where the first step is a selectable double-crossover event that deletes the target gene. We demonstrate how we have utilised this technique to delete two components of the proteinaceous shell of the *Geobacillus* propanediol-utilization microcompartment, and simultaneously introduce an oxygen-sensitive promoter in front of the remaining shell-component genes and confirm its functional incorporation.

**Conclusion:**

The selectable deletion of the target gene in the first step of our process prevents re-creation of wild-type which can occur in most homologous recombination techniques, circumventing the need for PCR screening to identify mutants. Our new technique therefore offers a faster, more efficient method of creating mutants in *Geobacillus*.

## Background

The *Geobacillus* genus comprises Gram-positive, metabolically-diverse thermophilic Bacilli formerly classified as group 5 [1]. It spans obligate aerobes, denitrifiers and facultative anaerobes. *Geobacillus thermoglucosidasius* NCIMB 11955 is a facultative anaerobe that can ferment C5 and C6 sugar monomers and oligomers via a mixed-acid pathway; it is therefore a potential candidate for commercial bioethanol and lactate production from lignocellulosic biomass, where, in addition to catabolic versatility, high fermentation temperatures reduce the cost of cooling, reduce the risk of contamination and facilitate easier removal of volatile products (e.g. ethanol). *G. thermoglucosidasius* has, therefore, been metabolically optimised to increase ethanol production through a series of gene knockouts and upregulations to create strain TM242 [2].

However, the limited availability of genetic tools means that chromosomal integration and deletion processes are problematic and slow, as commonly utilised techniques including lambda-red targeted deletions/integrations [3–5] and counter-selection with *sacB* [6] are unavailable at thermophilic growth temperatures. The most commonly used method to create directed chromosomal deletions or integrations in thermophiles involves the selection of antibiotic resistance markers inserted between flanking regions with homology to the target gene sequence using either a non-replicative vector or one with a temperature sensitive (T^s^) origin of replication [7–9]. However, with this method the antibiotic marker cannot be re-used because it remains at the site of integration. An alternative strategy, which was used to create TM242, places the antibiotic resistance marker outside the target flanking regions and utilises a two-step homologous recombination process, where the first step integrates the entire plasmid including the antibiotic resistance marker, and the second step relies on recombination via the second homologous flanking region that replaces the gene target and removes the marker along with the rest of the plasmid [10–13]. The disadvantage of this method is that there is no positive selection for the desired product, requiring lengthy passaging and screening for antibiotic sensitive colonies; also the re-creation of a wild-type strain is equally feasible producing the same phenotype, and therefore PCR screening of multiple colonies is required to confirm the presence of the desired knockout/insertion.

Counter-selection methods have been developed for thermophilic organisms to select for the second homologous recombination event and hence reduce the screening process. One such method is the *pyrF* based system [14, 15]. This technique utilises the toxicity to 5-fluoroorotic acid conveyed by the *pyrF* gene product to select for integration events. Although this method reduces the mutant selection process, it also necessitates the creation of a *pyrF* ^−^ *pyrR* ^−^ double-knockout parent strain and results in a final auxotrophic mutant; it would therefore be unsuitable for commercial strain production unless the mutations were repaired at the final stage. An alternative counter-selection system was described that utilized β-glucosidase (Bgl) and the synthetic substrate X-Glu (5-bromo-4-chloro-3-indolyl-β-d-glucopyranoside) [16]. This study demonstrated that an increase in the concentration of X-Glu led to a considerable reduction in the size of colonies; this was confirmed to be the result of toxic Bgl cleavage products of X-Glu by creation of a *bgl* deletion strain that showed no sensitivity to X-Glu. They demonstrated that it was possible to distinguish between strains carrying a plasmid with over-expressed Bgl and wild-type strains with just the native chromosomal copy of *bgl*, and this differential sensitivity was effectively utilised as a counter-selection tool for homologous integration events in *Thermus thermophilus* and *Micrococcus luteus* [16]. A similar approach has recently been reported for the moderate thermophile *Bacillus smithii* but using β-galactosidase as the counter-selection marker [17]. However, in both these studies PCR screening was still required to identify correct mutants.

Here we confirm that the X-Glu Bgl system can be used in *Geobacillus* spp. and describe a modified technique to create chromosomal deletions or integrations. This technique involves a two-step homologous recombination process where the first step involves a selectable double-crossover that deletes the target gene and incorporates an exchange allele and antibiotic resistance marker; the second crossover then excises the antibiotic resistance gene. This method not only creates marker-less mutations but also avoids the lengthy PCR screening process used with other homologous recombination techniques, because there is no possibility of re-creation of wild-type; all antibiotic sensitive colonies will possess the required mutation. This technique was implemented to delete *pduAB* in *G. thermoglucosidasius* TM242.

*Geobacillus thermoglucosidasius* encodes proteins for production of a propanediol-utilization (Pdu) microcompartment (MCP) necessary for B_12_-dependent metabolism of 1,2 propanediol; in other organisms Pdu MCPs have been shown to keep toxic/reactive intermediates isolated from the cytoplasm. The *Geobacillus* Pdu MCP is encoded by a 21 gene operon that comprises genes encoding a number of enzymes associated with the metabolism of 1,2 propanediol as well as a selectively-permeable proteinaceous shell [18]. As part of our studies to engineer the metabolism of *G. thermoglucosidasius*, we wanted to remove the Pdu MCP shell with the intention of releasing the Pdu enzymes into the cytoplasm where they would be available for a number of substrates taken up by the cell which are not imported into the Pdu MCP. A deletion of *pduAB* in *Salmonella enterica* has previously been reported to prevent formation of the MCP [19, 20], and therefore we targeted these genes with our new deletion strategy. The *pdu* operon is co-induced with the adjacent *cob* operon in response to 1,2-propanediol [21]; therefore to upregulate the metabolic enzymes in the remaining *pdu* operon and eliminate native regulation, the *pduAB* genes were replaced by a lactate dehydrogenase (LDH) promoter. Utilising our technique described here, the deletion and integration events were produced simply and efficiently.

## Methods

### Bacterial growth and culture conditions

The optimised ethanol-producing *G. thermoglucosidasius* TM242 strain (*ldh*A^−^ *pfl* ^−^ P_*ldh/pdh* ^up^) was utilised with the long-term goal of diverting the enhanced flux through acetylCoA to different reduced compounds. Cultures were grown in SPYNG media [1.6% (w/v) soy peptone, 1% (w/v) yeast extract, 0.5% (w/v) sodium chloride, pH 7] at 60 °C and 200 rpm, or on SPYNG medium agar (SPYNG medium plus 15 g/l agar) at 60 °C. Kanamycin was added to media or agar as appropriate at a concentration of 12.5 µg/ml. X-Glu was added to agar to a concentration described in the text.

For expression analysis, the modified *G. thermoglucosidasius* TM242 strain (*pduAB* ^−,^ *ldhp*) cultures were grown in modified Urea Salts Medium {USM; 0.5% (w/v) yeast extract, 2% (w/v) glucose, 25 mM NaH_2_PO_4_, 50 mM urea, 25 mM K_2_SO_4_, 5 mM citric acid, 3.125 mM MgSO_4_, 50 μM CaCl_2_, 2.5 μM Na_2_MoO_4_, and 12.5 ml/l of trace elements [60 mM H_2_SO_4_, 0.144% (w/v) ZnSO_4_·7H_2_O, 0.556% (w/v) FeSO_4_·7H_2_O, 0.169% (w/v) MnSO_4_·H_2_O, 0.025% (w/v) CuSO_4_·5H_2_O, 0.0562% (w/v) CoSO_4_·7H_2_O, 0.006% (w/v) H_3_BO_3_, and 0.0886% (w/v) NiSO_4_·6H_2_O], pH 6.7. The medium was buffered by adding 40 ml of each pre-sterilised buffer (stock concentration 1 M, pH 7.0) to bring the final concentration to 40 mM Bis–Tris, 40 mM HEPES, and 40 mM MOPS. The medium was also supplemented with 2.5 μM filter-sterilised biotin).

For qRT-PCR a colony of TM242 (*pduAB* ^−^, *ldhp*) was inoculated into 10 ml of SPYNG medium and grown at 60 °C overnight. One millilitre of this culture was then used to inoculate pre-warmed USM and cultures were grown under either aerobic (50 ml USM in a 250 ml baffled flask) or oxygen limited (15 ml in a 30 ml glass bottle sealed with a PTFE membrane) conditions at 60 °C with shaking at 250 rpm. The oxygen limited culture was harvested after 6 h growth at an OD_600_ of ~4.0 and the aerobic culture was harvested after 3 h growth at an OD_600_ of ~1.4. A 6 ml sample of each culture was combined with 20 ml RNA protect solution (Qiagen, Hilden, Germany) and incubated for 5 min at room temperature. Cells were then harvested by centrifugation at 4000×*g* for 10 min and the pellets frozen at −80 °C until required.

### Plasmid construction

All primers referred to are listed in Table 1. pUCG3.8Bgl: the *Thermus thermophilus* β-glucosidase gene (*bgl*) was codon harmonised utilising EuGene Genetic Optimisation software, synthesised by Life technologies GeneArt^®^, and supplied in the vector, pBAD. The *bgl* gene was amplified from the pBAD vector using primers Bgl_F and Bgl_R and then cloned into the *Geobacillus* expression vector pUCG3.8 [22], digested with *SmaI*, utilizing NEB Gibson Mastermix^®^ to create pUCG3.8Bgl.

**Table 1:** Primers used in the construction of plasmids

Oligonucleotide name	Sequence
Bgl_F	atgcctgcaggtcgactctagaggatccccacgggccagtttgttgaaga
Bgl_R	accatgattacgaattcgagctcggtacccttagctgcgatacgctccgc
Pdu-start_F	ggcgtctaacaggcgcgtagagacatggttcgagaagcattagg
Pdu-start_R	atattcccccgacttaaagacgctaaaatttctgtattggttgc
Pdu-5′_F	gcatcaggcgccattcgccattcaccagtcatccatattgctttag
Pdu-5′_R	cccggggcggccgcagctagcacgtggacctccctctacta
Pdu-3′_F	gctagctgcggccgccccgggtctttgggagggaatgttatgag
Pdu-3′_R	cttcccaacagttgcgcagcctcacggcctgtttgtgagccaac
LdhF	aaaaaagctagctcgcggagcggggaattaaaaag
LdhR	aaaaaacccgggcgctgtctgtcatcctttcc
Test_F	gaatgctttagaatatggag
Test_R	gagcttctagaaaatacag

*pduAB* deletion plasmid: for the ‘start region’, a 600 bp region was PCR-amplified from *pduA* bp 1 to *pduB* bp 311 using Pdu-start_F and Pdu-start_R primers, and then cloned into a *ZraI* digested pUCG3.8Bgl vector using the NEB Gibson Mastermix^®^ 140 bp upstream of the kanamycin resistance gene, creating pUCG3.8Bgl+start. For the ‘5′3′ flank’ region, two 600 bp regions upstream and downstream of the *pduAB* genes were PCR-amplified using Pdu-5′_F, Pdu-5′_R, Pdu-3′_F and Pdu-3′_R primers, and then joined by overlap extension PCR using Pdu-5′_F and Pdu-3′_R primers. The 5′3′ flank was then inserted into pUCG3.8Bgl+start region at the *bglI* site downstream of the kanamycin resistance gene, utilizing the NEB Gibson Mastermix^®^. To upregulate the rest of the metabolic enzymes of the *pdu* operon, the LDH promoter was cloned into the multiple cloning site in the 5′3′ flanking region. To do this, the LDH promoter was PCR-amplified from *G. thermoglucosidasius* NCIMB 11955 with ldhF and ldhR primers containing flanking *Nhe*I and *Xma*I restriction sites. The PCR product and pUCG3.8Bgl+start+5′3′ flank were digested with *Nhe*I and *Xma*I, gel purified and ligated with T4 ligase to create pUCG3.8Bgl-pdu. The ligation reaction mixture was transformed into *E. coli* DH5α cells and transformants selected on LB agar with kanamycin.

### Transformation and integration

*Geobacillus thermoglucosidasius* TM242 was made electrocompetent as described previously [23]. The cells were transformed with approximately 200 ng plasmid DNA; cells were electroporated at 1.75 kV, 600 Ω resistance and 25 capacitance, resuspended in 1 ml SPYNG medium and, after incubation with shaking for 1.5 h, were plated onto SPYNG medium agar with kanamycin. Plates were incubated overnight and colonies selected.

### Integration protocol

Transformant colonies were selected and grown overnight with shaking in 5 ml of SPYNG medium containing kanamycin. 1 ml of the overnight culture was sub-cultured into 20 ml of SPYNG medium with kanamycin and grown for a further 8 h to increase the chance of integration, then 100 µl of the culture were plated onto SPYNG medium agar containing kanamycin and 1 mg/ml X-Glu, and then incubated statically overnight. A single large colony was selected and re-streaked onto SPYNG medium agar with X-Glu and kanamycin to purify the integrant. The largest colony was selected and passaged four times for 4 h with shaking by serial sub-culturing 100 μl aliquots in 25 ml SPYNG medium with no antibiotic to allow the second crossover recombination to occur. 100 µl of the 4th passaged culture was plated onto SPYNG medium agar with no antibiotic and incubated statically overnight. Colonies were selected and replica plated onto SPYNG medium agar with and without kanamycin. Sensitive colonies were verified by PCR to confirm the presence of the required chromosomal deletion.

### qRT-PCR

To isolate RNA, frozen cell pellets were re-suspended in 250 µl of lysis buffer [30 mM Tris–HCl, pH 8.0, 1 mM EDTA, 15 mg/ml lysozyme (Sigma), 20µl Proteinase K (Qiagen)] and incubated for 10 min at room temperature, mixing every 2 min. RNA isolation and purification were performed using the RNAeasy Mini Kit (Qiagen), the concentration and purity were measured with a NanoDrop 2000 Spectrophotometer (Thermo Fisher Scientific, Waltham, MA, USA). For reverse transcription (RT), 800 ng of the isolated RNA was used to synthesize cDNA from random primers using a High Capacity cDNA RT Kit (Applied Biosystems). Triplicate RT reactions were performed for each RNA sample and the samples pooled. The resulting cDNA concentration and purity were measured with the NanoDrop 2000 Spectrophotometer (Thermo Fisher Scientific, Waltham, MA, USA).

To assess the changes in expression of the *pdu* operon using qPCR, two reference genes were selected: RNA polymerase β (*rpoB*) and β-prime (*rpoB*′) were chosen as *rpoB* has previously been identified as a housekeeping gene in *Bacillus* species [22] and no information on appropriate housekeeping genes in *Geobacillus* was available in the literature. Primers for the quantification of target and reference genes were designed using Primer3 software [23]. LuminoCt SYBR Green qPCR Readymix (Sigma Aldrich) was used for all qPCRs. Each reaction contained 10 μl of Master Mix, 0.3 μM of each primer, 4 μl of diluted cDNA template and ultrapure water to a final volume of 20 μl. qPCR was performed on Chromo4 Real time PCR detector, BioRad. The qPCR cycle consisted of an initial enzyme activation step at 95 °C for 20 s, followed by 40 cycles of denaturation at 95 °C for 5 s and annealing and extension at 60 °C for 30 s. A melt curve analysis was performed by raising the temperature at the end of each run by 0.2 °C per 2 s from 55 to 95 °C. Standard curves were created for each primer set with qPCR, using a dilution series of pooled cDNA from individually synthesized samples as a template.

## Results

### Toxicity of X-Glu to strains carrying pUCG3.8bgl

*Geobacillus thermoglucosidasius* is known to encode a β-glucosidase (Bgl) [22], which hydrolyses the synthetic substrate X-Glu to release an indoxyl product that dimerises to form the blue indigoid dye, 5,5′-dibromo-4,4′-dichloro-indigo. In *Thermus* and *Micrococcus* species this dye has been found to be toxic, making it useful as a tool for counter-selection strategies [16]. To establish whether the X-Glu hydrolysis product was toxic to *G. thermoglucosidasius*, strains TM242 and TM242 pUCG3.8bgl, containing the *T. thermophilus bgl* gene expressed from a strong constitutive *rpsL* promoter on the high copy number replicative plasmid pUCG3.8 [22], were plated on SPYNG agar with concentrations of X-Glu at 500, 700, 800, 1 and 1.2 mg/ml (data not shown). TM242 was able to grow in concentrations up to 1 mg/ml of X-Glu, with growth inhibited at 1.2 mg/ml. The strain TM242 pUCG3.8Bgl, constitutively expressing *T. thermophilus* Bgl, was only able to grow up to a concentration of 800 µg/ml X-Glu with growth inhibited at 1 mg/ml. Therefore, it was possible to distinguish between strains carrying and expressing the additional *bgl* gene from *T. thermophilus* from a high copy number plasmid and the unmodified TM242 by growth on plates containing 1 mg/ml X-Glu.

### Use of β glucosidase (Bgl) and X-Glu as a selectable marker system

**Fig. 1:**
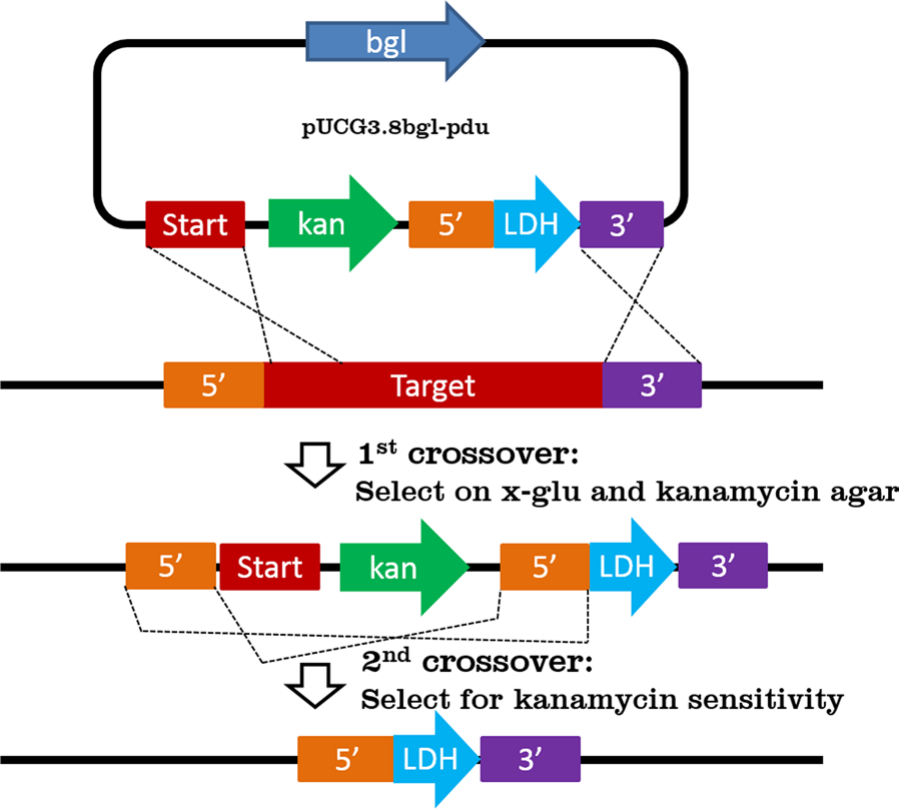
pUCG3.8Bgl-pdu *ldh* promoter integration protocol. The *ldh* promoter is inserted between the 5′ and 3′ flanking regions on pUCG3.8Bgl-pdu. The 1st crossover event relies on homologous recombination of the plasmid with two locations on the chromosome: the start of the target gene and the 3′ flanking region. The first double crossover will delete the target gene. This event is selected on X-Glu kanamycin agar, which only permits growth of cells that have the integrated construct with kanamycin resistance and have lost the plasmid backbone encoding the Bgl enzyme. The 2nd crossover event relies on homologous recombination between the 5′ flanking region of the target gene and the duplicate region incorporated from the plasmid in the 1st crossover event. This second crossover will render the cells sensitive to kanamycin and can be identified by replica plating

**Fig. 2:**
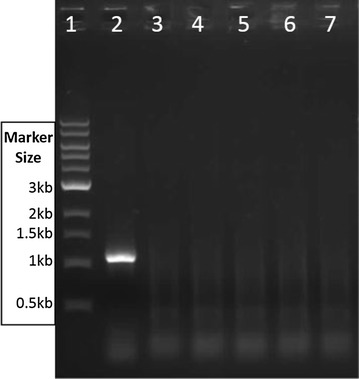
Colony PCR to confirm the first crossover event. Following re-streaking, five colonies were selected from agar with kanamycin and X-Glu, boiled in 20 µl milliQ water and PCR amplified with Bgl_F and Bgl_R. Following a double-crossover, the plasmid *bgl* gene would not be present. *Lane 1* Biolabs 1 Kb ladder, *lane 2* the 1.1 kb control *bgl* PCR product from unintegrated TM242 pUCG3.8Bgl-pdu, *lanes 3*–*7* no PCR product from colonies selected on kanamycin X-Glu plates

**Fig. 3:**
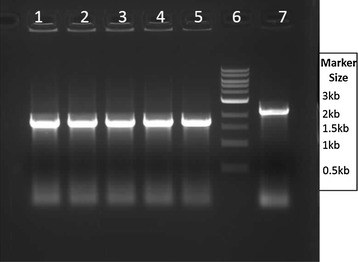
Colony PCR to confirm second crossover event. Colonies were selected, boiled and PCR-amplified with primers Test_F and Test_R, with homology to chromosomal regions just outside the flanking regions. *Lanes 1*–*5* kanamycin sensitive colonies selected following the second crossover event, showing a band size 1.58 kb confirming *pduAB* deletion. *Lane 6* Biolabs 1 kb marker. *Lane 7* a PCR result from an un-modified TM242 colony with a 2.2 kb band

The Bgl/X-Glu counter-selection system was implemented to delete the shell proteins of the Pdu MCP. This was undertaken to produce enzymes normally present within the Pdu MCP in the cytoplasm, thus removing the selectivity of the MCP shell [18]. The shell genes *pduA* and *B* were replaced with an *ldh* promoter to eliminate native regulation and upregulate the metabolic enzymes in the rest of the *pdu* operon, downstream of *pduB*. The plasmid pUCG3.8Bgl-pdu was electroporated into *G. thermoglucosidasius* TM242. Figure 1 shows the integration and deletion strategy. The first integration event was selected by growth on SPYNG medium containing kanamycin and 1 mg/ml X-Glu, which should counter-select for the un-integrated plasmid. If the incorporation of *bgl* gene into the chromosome from a single crossover event increased the production of the toxic indigoid dye to a level that was non-permissive, growth should only occur following a double crossover event incorporating the kanamycin resistance gene but not *bgl*. Although preliminary tests had used a high copy number plasmid, it was anticipated that use of the strong promoter *rpsL* would also result in sufficiently high *bgl* expression levels after chromosomal integration to confer toxicity to X-Glu at the concentration tolerated by the WT strain. Approximately 150 colonies were observed on the X-Glu and kanamycin plates, and were assumed to contain double-crossover integrations. A single, large colony was selected, re-streaked onto X-Glu and kanamycin agar for purification, and incubated overnight. Five colonies were selected and colony PCR was used to confirm the absence of the plasmid backbone, confirming that all colonies had lost the *bgl* gene, but as they were kanamycin resistant, presumably contained the desired double crossover integrations (Fig. 2). Therefore, our assumption that a single crossover incorporating a single copy of *bgl* expressed from a strong promoter would be toxic was confirmed. One of the colonies containing a double crossover integration was selected, passaged four times in SPYNG media containing no antibiotic to encourage the second crossover recombination, and then plated onto agar with no antibiotic added. Three hundred and fifty colonies were selected, replica-plated onto agar with and without kanamycin and incubated overnight. Sensitive colonies would occur following a second crossover event, creating a marker-less chromosomal target gene deletion mutant. Five colonies were found to be sensitive to kanamycin, and colony PCR confirmed that these sensitive colonies were indeed deletion mutants (Fig. 3).

### Confirmation of the integration of the *ldh* promoter

As well as gene deletion, the design of the pUCG3.8Bgl-pdu vector allows the substitution of the deleted gene(s) with a new gene/allele, by placing this gene immediately downstream of the second 5′ flanking region. In this experiment we needed to place the genes downstream of *pduB* under the control of a separate promoter so that they could be independently expressed. In this instance we used the *ldh* promoter, which is induced under oxygen limitation. In order to assess its efficacy we measured expression of the downstream genes *pduC*, *D* and *E* in cultures that were grown under aerobic and oxygen-limited conditions [22], by qRT-PCR. Aerobic cultures were grown in 50 ml media in 250 ml volume flasks, whilst oxygen-limited cultures were grown in 15 ml media in a 30 ml tube sealed with a PTFE membrane; the cultures samples were frozen with Qiagen RNAprotect. A qPCR screen was set up as described using 5 ng cDNA for each condition (aerobic or oxygen-limited [22]) with each primer pair (target or reference) (Table 2) in triplicate. The average C(t) value was calculated for each condition and primer, and the ratio of expression between the aerobic and oxygen limited conditions was determined using the Pfaffl equation [24]. The results of this analysis are shown in Table 3. Consistent with the known expression of the *ldh* promoter, the *pduC*, *D* and *E* genes were all expressed to a higher level under oxygen limited conditions compared to oxygen excess; the *pdu* genes were upregulated between 5.4- and 8.1- fold based on using *rpoB* as the reference reference gene, but between 19.5- and 29.5- fold when *rpoB*′ gene expression was used (Table 3). While both data sets clearly show the upregulation of the *pdu* operon by the *ldh* promoter under oxygen limited conditions the large numerical differences obtained using different reference genes warranted some investigation. In *G. thermoglucosidasius rpoB* and *rpoB*′ are part of an operon with *rpoB*′ downstream of *rpoB.* Although the intergenic region did not reveal any evidence for transcriptional regulators a potential rho-independent transcriptional terminator (CCAAGCCGCATGGGGCTT) was found within the early coding region of the *rpoB*′ gene, as identified by the online tool “ARNold” (http://rna.igmors.u-psud.fr/toolbox/arnold/). This suggests that expression of *rpoB*′ could be under the control of physiologically-dependent attenuation which would result in differential expression compared to *rpoB*. If transcriptional termination was more frequent under oxygen limited conditions than under fully aerobic this would explain the much higher apparent ratios obtained when using *rpoB*′ as a reference gene.

**Table 2:** Primers used for qRT-PCR

Gene	Primer	Sequence
*pduC*	Fwr	ccatatcgctccacgaaaga
	Rev	ctgcagcttcccggactaa
*pduD*	Fwr	tgccggtattgaagaagagg
	Rev	cgtcgttcctttggactgaa
*pduE*	Fwr	cgtattgcagcgcgattt
	Rev	gtggctgcttcgacttcttc
*rpoB*	Fwr	ctcttggctttggctctgac
	Rev	gacgcaaacgctcgtaaatc
*rpoB*′	Fwr	caagagcattgacggatgg
	Rev	cccgtttctggatgtctcac

**Table 3:** Ratio of target gene expression under the control of an integrated *ldh* promoter (*pduC,* Pdu dehydratase large subunit; *pduD*, medium and *pduE* small subunit) under oxygen limited: aerobic growth conditions

Target	Reference	Ratio of target gene expression in oxygen limited/aerobic conditions
*pduC*	*rpoB*	8.1
*pduD*	*rpoB*	5.4
*pduE*	*rpoB*	7.6
*pduC*	*rpoB*′	29.5
*pduD*	*rpoB*′	19.5
*pduE*	*rpoB*′	27.5

## Discussion

Gene deletion in *Geobacillus* spp. typically utilises a two-step process involving initial integration of the entire deletion vector, aided by the use of a temperature-sensitive replicon to select for loss of the independently replicating host plasmid, followed by selection for a second homologous cross-over event. The second step requires extensive screening and, although technically a second homologous recombination event should have a 50% chance of yielding mutants and wild-type (assuming the flanking regions of homology are of similar size), the re-creation of wild-type is frequently found to predominate. The use of a counter-selection marker, which provides positive selection for this second recombination event, eliminates the need to screen for loss of an antibiotic resistance gene (negative selection), but still does not alter the bias towards recovering wild-type which presumably reflects physiological counter-selective pressure.

In this study we describe the development of an efficient selection system to create marker-less chromosomal deletions and gene/allelic replacements in *Geobacillus* spp. which eradicates this bias by allowing direct selection for complete gene deletion as the first step, thus removing the possibility of re-creation of wild-type. The system utilises kanamycin resistance (kanamycin is the most thermostable of the common antibiotics currently in use) as a positive selection marker and the expression of β-glucosidase (Bgl) which converts the synthetic substrate X-Glu into a toxic indigoid dye, as a negative selection. The design of the vector means that the integration process incorporates a selectable double-crossover event as the initial step, which deletes the target gene. By using a two-step transformation and subsequent chromosomal integration, it was possible to obtain over 100 probable double homologous cross-over colonies from 100 μl of a liquid culture. In this study, all the kanamycin and X-Glu resistant colonies selected had the desired double cross-over. These would have been unable to yield wild-type genotype, an event that can occur in other systems involving two-step homologous recombination [10–16]. The final step involves elimination of the integrated kanamycin resistance gene, which does require screening (although it would also be possible to integrate a second counter selection marker with the kanamycin resistance gene). However, elimination can only occur through recombination between the two 5′ repeat regions. Thus, 100% of the kanamycin sensitive colonies recovered contained the desired gene knockout. Therefore, our method offers a quicker, simpler process eliminating the need for PCR screening of mutant colonies.

The *bgl* gene with X-Glu was found to be an effective counter-selection system in *G. thermoglucosidasius*. We demonstrated that, although TM242 showed sensitivity to X-Glu, presumably due to cleavage by the native Bgl enzyme, it was able to tolerate a concentration of 1 mg/ml X-Glu on agar plates. At this concentration the TM242 pUCG3.8bgl strain over-expressing β-glucosidase was unable to grow. It follows that, in a mixed culture of an untransformed strain and a strain carrying pUCG3.8bgl, only the strain lacking or expressing low levels of Bgl would be able to grow on agar with 1 mg/ml X-Glu. The vector incorporates three non-contiguous regions of homology to the chromosomal target to facilitate the initial deletion of the target gene and subsequent resolution to remove the kanamycin resistance marker (Fig. 1). A simple version in which the 5′ and 3′ flanking regions are fused (e.g. by overlapping PCR) may be used for gene deletion. However, the construct also allows for gene replacement as an additional outcome. To demonstrate the efficiency of this we utilised this system both to delete *pduAB* and to replace them with an *ldh* promoter in TM242. The *ldh* promoter is upregulated under oxygen-limited conditions to allow lactate production as an overflow pathway when the activity of the respiratory chain becomes limited. This precedes the activation of the full fermentation pathway which produces formate, acetate and ethanol. Using qRT-PCR we demonstrated that expression of the *pduC*, *D* and *E* genes were all upregulated to a similar extent under the control of the inserted *ldh* promoter which is induced under oxygen limitation, consistent with the evidence that the 17 *pdu* genes form a large operon. Different degrees of upregulation were recorded using *rpoB* and *rpoB*′ as reference genes which we suspect is due to transcriptional termination of *rpoB*′ expression under oxygen limited conditions. This would change the ratio of *rpoB* and *rpoB*′ transcripts despite their presence in an operon. We, therefore, recommend that, out of the two, *rpoB* is used for future qRT-PCR analyses.

This new technique has advantages over the commonly utilised two-step homologous recombination approach where an antibiotic resistance marker is first integrated and then removed together with the targeted chromosomal DNA. The double-crossover strategy deletes the target chromosomal gene in the first step. Assuming this is not lethal, it removes any counter-selective pressure to select for the re-creation of the wild-type genotype, which is commonly encountered with the two-step approach. Indeed, wild-type re-creation is no longer possible with these mutants so the second homologous recombination to eliminate the kanamycin resistance marker can only yield the desired product, obviating the need for PCR screening. This method does not require the introduction and subsequent removal of auxotrophic mutations and, as it does not leave any residual markers, can be used repeatedly for strain engineering.
